# 

**Published:** 1861-04

**Authors:** 


### CONTENTS.

| ART. | | PAGE |
| --- | --- | --- |
| | Quarterly Retrospect . . . . . . . . . . . . . | xxiii—xxx |
| I. | On the Moral Phenomena of Insanity and Eccentricity. By Thomas Mayo . . . . . . . . . . . . . . . . . | 173 |
| II. | The Classic Land of Suicide . . . . . . . . . . . . | 182 |
| III. | Professional Tricksters . . . . . . . . . . . . . . . | 200 |
| IV. | Quarantine and the Spread of Epidemic Diseases. By Gavin Milroy, M.D., F.R.C.P. . . . . . . . . . . . . . . | 205 |
| V. | Cottage Asylums. By W. A. F. Browne, Commissioner in Lunacy for Scotland . . . . . . . . . . . . . . . | 213 |
| VI. | Modern Surgery . . . . . . . . . . . . . . . . . | 238 |
| VII. | The Deformed and their Mental Characteristics . . . . . | 246 |
| VIII. | On the Structure of the Inductive Syllogism and its Correlation to the Deductive. By R. G. Latham, M.D., F.R.S., &c. . . | 253 |
| IX. | Metanoia:—A Plea for the Insane. By Henry McCormac, M.D. | 262 |
| X. | Special Hospitals . . . . . . . . . . . . . . . | 271 |
| XI. | Honoraria . . . . . . . . . . . . . . . . . . | 275 |
| XII. | Physiology and Legislation . . . . . . . . . . . . | 285 |
| XIII. | Miscellanea Medica . . . . . . . . . . . . . . . | 293 |
| XIV. | The "Art of Rising" in Physic . . . . . . . . . . . | 303 |
| XV. | Cellular Pathology . . . . . . . . . . . . . . . | 311 |
| XVI. | Medical Gossip . . . . . . . . . . . . . . . . | 319 |
| XVII. | Literary Gossip and Record . . . . . . . . . . . . | 323 |
| XVIII. | In Memoriam:—William Baly . . . . . . . . . . . . | 334 |
| | Foreign Medico-Psychological Literature . . . . . . . | 341 |

No. III. will be published on the 1st of July.

